# Gastric Ultrasound for Gastric Content Evaluation

**DOI:** 10.4274/TJAR.2023.231479

**Published:** 2023-12-27

**Authors:** Shubha Srinivasareddy

**Affiliations:** 1 Penn State Milton S. Hershey Medical Center, Consultant in Anaesthesia and Chronic Pain, Pennsylvania, United States

**Keywords:** Gastric content, ultrasonography, preoperative fasting, residual gastric volume

## Abstract

Gastric content aspiration occurs once every 2000-3000 general anaesthetics. It is associated with a 20% incidence of in-hospital mortality. The incidence of pulmonary aspiration in patients undergoing surgery is at least three times more, up to 1 in 895 general anaesthetics. Pulmonary aspiration indeed is associated with half of our airway-related mortality linked with anaesthesia. The pulmonary aspiration causes significant morbidity including respiratory failure, acute lung injury, and multi-organ failure in adults. This review study aims to compare the stomach volume and contents in patients following standard fasting guidelines by Point of care gastric ultrasound measurements. Perioperative gastric ultrasound is a developing diagnostic modality that is modest, easy, non-invasive and efficient. It is very helpful to determine gastric contents in adult, obese, paediatric, and obstetric patients. It is a dependable and replicable tool that can be used for effective anaesthetic management. Gastric ultrasound is an irreplaceable procedure to complement the use of fasting guidelines, particularly when these guidelines have not been followed, or may not be relevant. Further series of research with metanalysis is required to understand the influence of point-of-care gastric ultrasound assessment on perioperative outcomes.

**Main Points**

• Predictable pre-operative fasting status assessment.

• Performing successful gastric ultrasound study.

• Tips for optimal point of care ultrasound study.

## Introduction

As a well-trained anaesthesiologist, we do good perioperative assessment and examination of a patient.^1^ It is a chance to conduct a systematic survey of health systems and do a clinical examination, and to discuss any related issues of the procedure with the patient.^2^ There should be a discussion about the necessities for fasting, and in the theatre waiting area. It is important to confirm the patient has fasted as per the guidelines and is adequately fasted.^3^ Aspiration is associated with increased perioperative adverse outcomes with an increased gastric volume, acidity or particulate matter.^4^ Drugs used during anaesthesia causes absent or reduced airway reflexes and risk of aspiration of gastric contents. We have seen studies which showed that 1 in 2000-3000 anaesthetised patients carry risk of aspiration.^5^ Substantial morbidity and mortality occurs in 1:200 and 1 in 72,000-100,000 patients subsequently. Adverse events related to aspiration mainly occur during anaesthesia induction, but these events can occur during extubation and even intraoperatively.^6^

National Audit Project-4 revealed that in the hospitals in UK, aspiration was accountable for 50% of deaths and aspiration was the most common cause of death linked to airway complications.^7^ In Australia, 30% of aspiration cases were admitted in high dependency unit and of those, 4% died according to the Australian Anaesthetic Incident Monitoring Study. Anaesthesia Closed Claims Project in the US suggested that 57% of cases aspirated and resulted in death and 14% in permanent disability.^8^

With the above statistically evidence, it is of consideration to the anaesthesiologist to know what to expect regarding the gastric content’s nature and gastric emptying time. Gastric emptying time can be measured by various methods, namely paracetamol absorption, radiological studies, gastric ultrasound, and gastric aspirates.^9^

All the above study techniques are subjective assessment of gastric emptying time and gastric volume in fasted patients and they vary vastly from patient to patient. Peri-operative gastric ultrasound has emerged as a reliable tool for assessment of gastric contents as empty, clear fluid and solids, and when contents are clear fluid, to quantify the gastric volume.^10^ Gastric ultrasound has been widely used in anaesthesia education and practice. It has been considered as a valid and reliable tool in a variety of patient populations like severely obese individuals, pregnant and non- pregnant adults as well as paediatric patients.^11^

Assessing and measuring gastric content, volume, and transit time is crucial. While numerous invasive methods exist, such as evaluating paracetamol absorption, utilizing electrical impedance tomography, employing radiolabeled diets, conducting polyethylene glycol dilution studies, or suctioning gastric content through tubes, these approaches are invasive, time-consuming, and are not utilized in perioperative practice.^12^

### Anatomical Concepts

The stomach comprises five distinct parts: the cardia, fundus, body, antrum, and pylorus. Identifying the gastric antrum in the epigastric region through ultrasound is straightforward. Positioned as the most dependent section of the stomach, the gastric antrum facilitates the descent of gastric contents into this region.^13^ The gastric antral wall is characterized by five discrete layers-mucosa, muscularis mucosae, submucosa, muscularis propria, and serosa. While these layers are not clearly visible on ultrasound, they are arranged from luminal to extra-luminal. The gastric antrum is situated posterior and inferior to the medial margin of the left lobe of the liver, anterior to the tail of the pancreas, and adjacent to the aorta.^14^

### Acquisition of Images

### Patient Positioning

The epigastric region needs should be fully exposed. Gastric antrum is located in both supine and right lateral decubitus (RLD) positions. Significant volumes of gastric content are easily observable in the gastric antrum, while smaller amounts may persist in the gastric fundus. The supine position, due to the greater dependency of the gastric fundus, makes it challenging to visualize its contents. In contrast, RLD position facilitates the gravitational drainage of gastric content toward the antrum.The RLD position increases the sensitivity of ultrasound to detect smaller volumes. Hence the best position to visualise and check antral content is RLD position. While some suggest conducting gastric sonography with the patient in a semi-recumbent position, this method is less precise than using RLD position for quantifying gastric volumes. Applying RLD positioning can be impractical for certain patients, such as those who are critically ill, experiencing trauma, or are pregnant. In such cases, scanning in the semi-recumbent position serves as a practical alternative (Table 1).

### Transducer Criteria

It is essential to measure a good clinical surface area. A convex probe (1-5 MHz) transducer is used. Sufficient penetration of the abdominal compartment is required to produce good sonographic images of the key landmarks. With regards to low BMI and paediatric patients, a linear, high-frequency (5-12 MHz) probe can be used to provide better visualisation of the superficial antrum and surrounding structures.^15^ The sonographic gel is applied on the probe to work as an acoustic medium. Changes in the depth and gain has to be performed according to individual patient body habitus to appropriately visualise the gastric contents.^15,16,17^

### Ultrasound Imaging Technique

**Figure 1:**
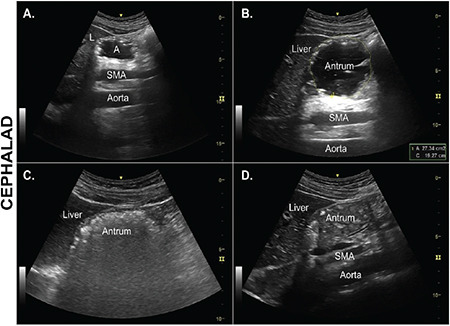
Ultrasound representation of gastric antrum.

Ultrasound transducer is placed in a sagittal plane in the epigastric region, immediately below the xiphisternum to visualise the gastric antrum. Conventionally, the transducer orientation positions cephalad to the left of the screen. The ultrasound machine’s probe is aligned vertically to the skin and is swept horizontally from the left costal margin to identify key sonographic landmarks in a sequence from deep to superficial. These include the vertebral bodies, abdominal aorta, head or neck of the pancreas, inferior margin of the left lobe of the liver, and the gastric antrum (Figure 1).^18^ The gastric antrum is observed through the acoustic window formed by the liver, allowing differentiation from other hollow viscera like the duodenum or bowel. Identification is facilitated by the antrum’s thick, hypoechoic muscularis layer, along with the hyperechoic serosa and mucosal layers, typically measuring 4 mm in thickness (Figure 1), and its superficial anatomical location. The antrum proves to be the most accessible part of the stomach for sonography, yet achieving a complete observation can be challenging due to the presence of air in the stomach body. Obtaining the optimal sonographic window may necessitate sliding the transducer from left to right or right to left to visualize the antrum in the short axis at the level of the aorta.^19^ To minimize obliquity, maneuvers such as heel-to-toe movements or transducer rotation can be employed to obtain clear antral views.^20^

## Discussion

The concept of full stomach has been universally followed to protect against vomiting, regurgitation and aspiration during anaesthesia. However, stomach can never be completely empty, since it continues to secrete gastric fluid, even after overnight fasting. Studies have shown that prolonged fasting has been associated with reduced gastric pH due to increased gastric acid secretion and increase in gastric volume, placing the patients at risk category for aspiration. Prolonged fasting in preoperative period is known to increase the risk of dehydration, vomiting and anxiety.

Preoperative gastric fluid volume measurement done after the aspiration of gastric contents using Salem sump tube following ingestion of 150 mL of clear fluids 3 hrs before ambulatory surgery did not show any significant volumes. Hence it was concluded that healthy patients can be allowed to drink clear fluid until 3 hrs before surgery. Other techniques which have been traditionally used to assess the contents and volume of the stomach include paracetamol absorption, electrical impedance tomography, radio-labelled diet, polyethylene glycol dilution and imaging techniques like scintigraphy and MRI. These tools may not be used in the acute setting and with the advent of bedside gastric ultrasonography, the gastric contents and volume can be assessed easily in the perioperative period. Gastric ultrasonography has become an indispensable tool in anaesthetic practice and has been proven to be as reliable as gastric scintigraphy with Tc99m, which is considered the gold standard for the assessment of gastric volume.

Ultrasound has been the first non-invasive technique that provides both quantitative and qualitative information about the gastric contents and its volume. Numerous mathematical models have been devised to calculate gastric volume by utilizing ultrasonographic images of the gastric antrum and computing its cross-sectional area. Perlas et al.^15^ introduced a precise linear model, derived from gastroscopic fluid assessment, demonstrating a mean difference of 6 mL between the predicted and measured volumes. It was applicable to adult, non-pregnant subjects with BMI up to 40 kg m^2^ and can predict volume up to 500 mL. The ultrasonographic images were used to categorise the gastric antrum into 3 grades depending on the presence of liquids as grade 0 - empty antrum in both supine and RLD positions, grade 1 - presence of liquid in RLD only and grade 2 - presence of liquid in both RLD and supine positions. The images were recorded after overnight fasting and then 2 hrs after ingestion of 200 mL and 500 mL isotonic solution. The gastric antrum was easily identified, once we located the left lobe of the liver and aortic/superior mesenteric artery pulsations.

### Study Limitations

The ultrasonographic evaluation of gastric volume being a subjective assessment, the range of results depends upon the skill of the assessor. Hence there might be subjective variations in the results. Gastric emptying is also affected by pain, anxiety and use of preoperative medications. These criteria were not addressed and could be a limitation of the study.

## Conclusion

Point of care gastric ultrasound proves invaluable in aligning with nil per oral guidelines, especially in situations where these guidelines may not have been adhered to or may not be suitable. However techniques should be improved to warranty better visualisation and assessment.

### Tips for Optimal POCUS Study

The gastric antrum is found superficially posterior to the rectus muscle, immediately adjacent to the left lobe of the liver and anterior to the pancreas and great vessels (Figure 1).

The thoracic spine may be seen posterior to the great vessels, particularly in slim subjects or children.

Critical identifying features of the stomach, which can help differentiate it from other hollow viscus, are a multi-layered wall (though not all five layers are typically visualized with a curvilinear probe) and the consistent location adjacent to the liver edge with the great vessels, preferably the aorta, in the far field of the image.

Because peristalsis can dramatically change the antral size from second to second, it is important to view the antrum for at least 10-15 seconds to obtain a representative observation.

## Figures and Tables

**Table 1:**
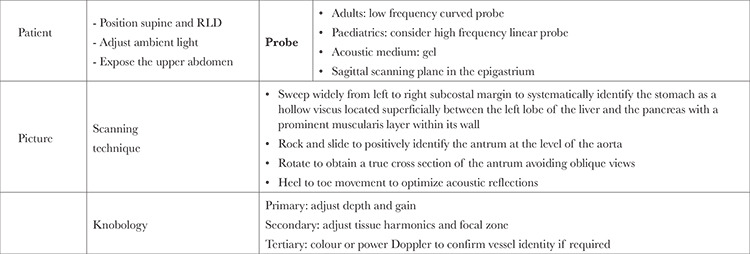
Successful algorithm for a good POCUS study^21^
